# Impact of depression on speech perception in noise

**DOI:** 10.1371/journal.pone.0220928

**Published:** 2019-08-15

**Authors:** Zilong Xie, Benjamin D. Zinszer, Meredith Riggs, Christopher G. Beevers, Bharath Chandrasekaran

**Affiliations:** 1 Department of Hearing and Speech Sciences, University of Maryland, Maryland, United States of America; 2 Department of Linguistics and Cognitive Science, University of Delaware, Newark, Delaware, United States of America; 3 Department of Communication Sciences and Disorders, The University of Texas at Austin, Austin, Texas, United States of America; 4 Department of Psychology, The University of Texas at Austin, Austin, Texas, United States of America; 5 Institute for Mental Health Research, Austin, Texas, United States of America; 6 Department of Communication Science and Disorders, School of Health and Rehabilitation Sciences, University of Pittsburgh, Pittsburgh, Pennsylvania, United States of America; University of Hull, UNITED KINGDOM

## Abstract

Effective speech communication is critical to everyday quality of life and social well-being. In addition to the well-studied deficits in cognitive and motor function, depression also impacts communication. Here, we examined speech perception in individuals who were clinically diagnosed with major depressive disorder (MDD) relative to neurotypical controls. Forty-two normal-hearing (NH) individuals with MDD and 41 NH neurotypical controls performed sentence recognition tasks across three conditions with maskers varying in the extent of linguistic content (high, low, and none): 1-talker masker (1T), reversed 1-talker masker (1T_tr), and speech-shaped noise (SSN). Individuals with MDD, relative to neurotypical controls, demonstrated lower recognition accuracy in the 1T condition but not in the 1T_tr or SSN condition. To examine the nature of the listening condition-specific speech perception deficit, we analyzed speech recognition errors. Errors as a result of interference from masker sentences were higher for individuals with MDD (vs. neurotypical controls) in the 1T condition. This depression-related listening condition-specific pattern in recognition errors was not observed for other error types. We posit that this depression-related listening condition-specific deficit in speech perception may be related to heightened distractibility due to linguistic interference from background talkers.

## Introduction

Depression is a leading cause of disability worldwide [1]. It is characterized by impairments in cognitive, psychomotor speed, and speech communicative behaviors [2,3]. To date, however, communicative behaviors remains the least characterized deficits in depression, despite the fact that effective communication is critical to social well-being and communication deficits may exacerbate depressive symptoms [4]. Extant work on speech communication has mainly focused on speech output in individuals with depression [5]. For example, verbal fluency is shown to be reduced in individuals with major depressive disorder (MDD) [3]. Speech rates in individuals with MDD are predictive of depression severity as well as response to treatment [6]. Relative to the rich literature on speech production, much less is known about speech perception in individuals with MDD. Hence, the current study aims to examine the effect of depression on speech perception in conditions that mimic everyday listening environments.

In typical communication situations, speech perception is often affected by the speech of other unattended talkers (often referred to as a ‘cocktail party’ situation [7]). Speech maskers contain linguistic information that is highly confusable with the target speech [8,9]. For successful speech perception, listeners are required to segregate the target speech from the mixture of acoustic inputs (i.e., object formation [8]); and to exert top-down attention to select the target speech and inhibit the interference from the speech maskers (i.e., object selection [8,9]). In contrast to speech maskers, other sources of noise may contain limited linguistic information (non-speech ‘energetic’ maskers), but can still disrupt speech perception [9–14]. For example, noise from construction sites or airplanes does not contain linguistic maskers but can still impact perception. In the extant literature, the two types of noise interference are distinguished as ‘informational’ masking and ‘energetic’ masking [15,16]. Speech maskers are posited to produce informational masking, in addition to energetic masking, while non-speech maskers produce relatively greater energetic interference. Recent work suggests that, in addition to informational and energetic masking, noise can compromise speech perception due to a third form of masking, i.e., modulation masking [17,18].

Hearing impairment (HI) and aging are two widely studied factors that can independently impoverish a listener’s ability to understand speech, particularly in the presence of interfering talkers [19–23]. Interestingly, HI is also associated with higher levels of depression [24–27]. For example, Li et al. (2014) reported that the prevalence of developing moderate to severe depression increased by 5.5% for adults with self-reported HI relative to those without HI. Depression is also common in older adults [28–31], and the prevalence of MDD for adults 65 and older ranges from 1% to 5% in large-scale samples from the United States [28]. Hence, the current investigation supports depression as a factor that can affect speech perception in noise (SPIN) independent of HI or aging. This suggests a critical need to separate out the unique mechanisms of SPIN deficits associated with HI, aging, and depression.

Chandrasekaran et al. (2015) examined the relationship between depression and SPIN in a nonclinical population. They found that normal hearing (NH) young adults with self-reported elevated depressive symptoms show a deficit of speech perception in conditions with speech maskers, but not with non-speech maskers. Hence, the first goal of this study was to replicate the effect of depression on SPIN in a clinical population. Specifically, we compared performance on SPIN in NH adults with a clinical diagnosis of MDD relative to a carefully matched group of neurotypical NH individuals. Prior work suggests a close link between sub-clinical elevated depressive symptoms and MDD, such that individuals with elevated depressive symptoms have a higher probability of developing MDD [32,33]. Considering these findings, we predicted that individuals with MDD would also exhibit a selective speech perception deficit in speech maskers but not in non-speech maskers.

Additionally, this study examined speech recognition errors from the SPIN data to understand the mechanisms underlying the hypothesized depression-related listening condition-specific (i.e., speech maskers) deficit in speech perception. In the literature related to SPIN, there is always interest in the examination of the errors in speech recognition produced by listeners, though a limited number of studies have actually implemented speech recognition error analyses [34–43]. The analysis of speech recognition errors can provide information not only about whether a listener recognizes words, but also about how the degraded speech signals are perceived and resolved by the listener [34]. Thus, the speech recognition error analysis is potentially useful in revealing the mechanisms underlying a listener’s speech perception performance [43].

In the current analysis of speech recognition, we first characterized the occurrence rates of whole sentence omission error, which is operationalized as a participant’s response that did not contain any of the content words from the target sentence. For this type of error, we further characterized whether the participant’s response contains content words of a distractor (masker) sentence. If we did not observe the whole sentence omission error, we then characterized the occurrence rates of another two error categories: word-level errors, i.e., substitution, addition, or omission of content words (nouns, verbs, adjectives, adverbs) and function words (closed-class) in the target sentence; and morpheme-level errors for content words in the target sentence (e.g. tense change, pluralization). In a previous study [43], error rates at all three of these levels (whole sentence, word, and morpheme) significantly differed between native- and non-native listeners across a variety of mask types. This finding suggests that linguistic processes can be affected by noise at multiple levels, which may be distinguishable by detailed error analysis.

Previous studies suggest increased susceptibility to distracting information in individuals with MDD [44–47]. Hence, we predicted that the occurrence rate of errors as a result of interference from the masker sentences would be increased in individuals with MDD (relative to neurotypical controls), particularly in conditions with speech maskers that contain highly distracting linguistic information [8,9].

## Materials and methods

### Participants

**Fig 1:**
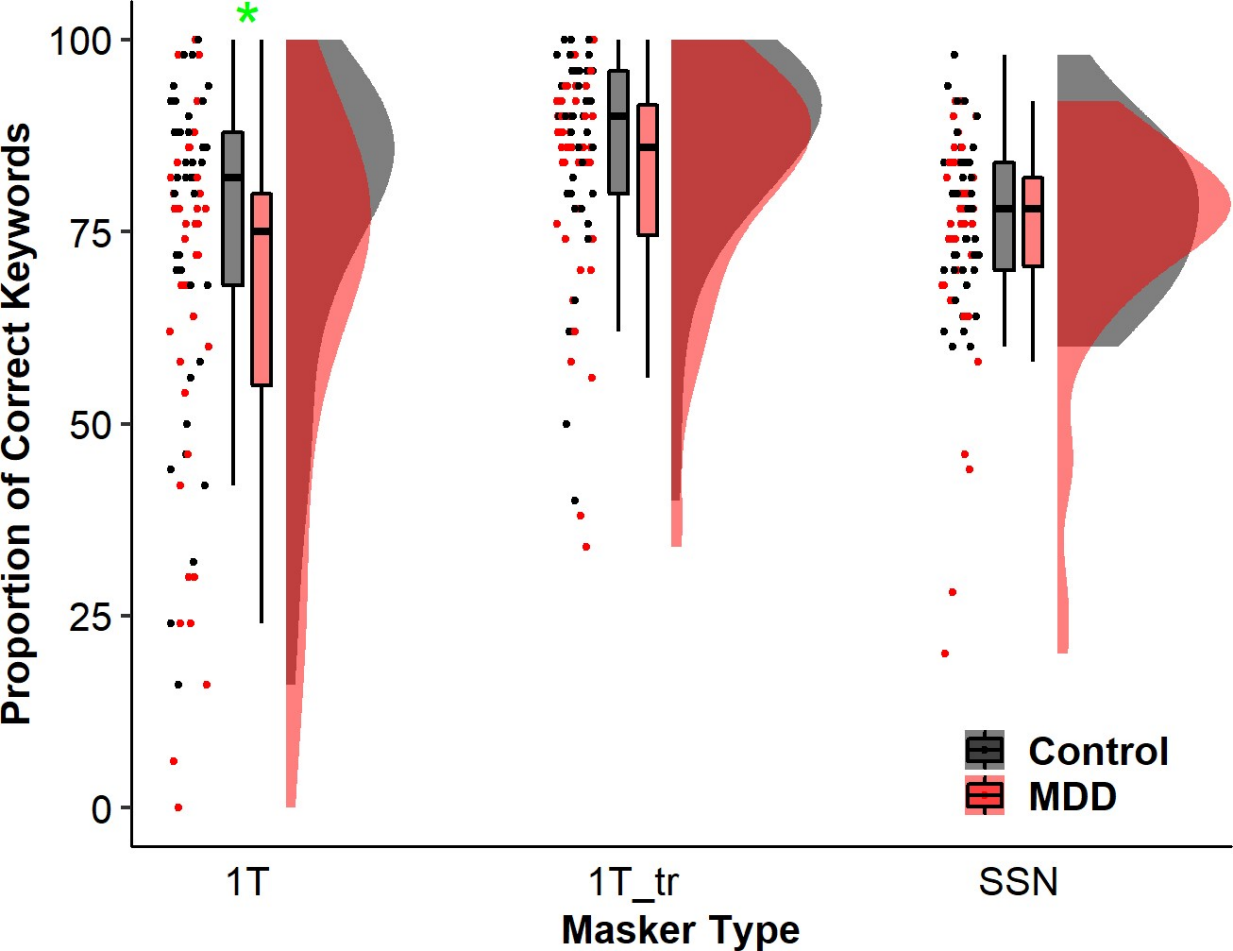
Raincloud plots (from left to right: jittered raw data for all participants, boxplots, and probability distribution of the data) of proportion of correctly identified keywords for neurotypical controls (black) and participants with MDD (red) across three types of masker: 1T (1-talker masker), 1T_tr (reversed 1-talker masker), and SSN (speech-shaped noise). For the boxplots, the boxes and the horizontal line inside show the quartiles (1st to 3rd quartile) and the median, respectively. The whiskers denote 1.5 times the interquartile range. Outliers, defined as cases with values outside the 1.5 interquartile range, were not displayed in the boxplots. * denote *p* < 0.05.

The present experiment is part of a larger project that examined emotion and cognition in major depression. Fifty-two patients with MDD and 51 neurotypical control participants were recruited from the greater Austin community. Three participants in the MDD group were excluded from the analyses because of missing data on the speech in noise task due to a software failure. Inclusion criteria for the individuals with depression were a DSM-V diagnosis of MDD by a trained native English research assistant using the structured Mini-International Neuropsychiatric Interview [48] and a score ≥ 16 on the Center for Epidemiological Studies Depression Scale (CES-D) [49] at the time of study. Note that those individuals with comorbid anxiety were not explicitly excluded from the study. Comorbid anxiety was determined as a DSM-V diagnosis of an anxiety disorder in addition to MDD using the Mini-International Neuropsychiatric Interview [48]. Inclusion criteria for the healthy control participants were no history of MDD and a score < 16 on the CES-D [49] at the time of study. One participant from the MDD group and five participants from the control group were excluded from analysis due to not meeting the CES-D criteria. Additional inclusion criteria for all participants included: age between 18 and 50 years, had normal or corrected vision, and being fluent in English via self-report. The self-reported fluency in English was further confirmed as having no difficulty in completing the screening surveys via phone, by a native English research assistant. One participant from the control group was excluded because of disfluency in English. We did not collect detailed information about language experience and proficiency, e.g., whether a listener is a monolingual or bilingual speaker of English. may confound our results. However, we believe such factor (e.g., bilingualism) may not be a factor influencing our results because the performance under conditions with non-speech maskers is comparable across groups (see Fig 1). Exclusion criteria for all participants were a current or past DSM-V diagnosis of psychosis, mania, alcohol dependence, alcohol abuse, or substance dependence.

All participants underwent a hearing screening. Five participants from the MDD group and four participants from the control group were further excluded for failing to meet the hearing thresholds, i.e., ≤ 25 dB hearing level for octave frequencies from 250 and 4,000 Hz for each ear. One participant from the MDD group was excluded because of incomplete data on the hearing screening.

The final sample for analysis consisted of 42 MDD participants and 41 control participants. Table 1 displays their demographics. As shown in Table 1, the MDD participants were matched as closely as possible with the control participants for age, and the ratios of sex, race, and ethnicity. All participants gave written informed consent and received monetary compensation under a protocol approved by the Institutional Review Board at the University of Texas at Austin.

**Table 1:** Demographics of the samples for analyses.

	MDD	Control	*p*
N	42	41	-
Age (M, SD, min/max)	23.76, 5.84, 18/46	24.73, 7.51, 18/49	.513^c^
Sex (female/male/other)	33/7/2	29/12/0	.179^d^
Race (Caucasian/other/unspecified)	19/23/0	21/19/1	.582^d^
Ethnicity (Hispanic/Non-Hispanic/unspecified)	10/31/1	6/34/1	.698^d^
Age onset for 1^st^ depressive episode (M, SD, min/max)	16.8, 4.78, 11/29^a^	NA	-
Number of depressive episode (single /recurrent/unspecified)	9/28/5	NA	-
Currently taking medication	7	0	-
Currently in therapy or counseling	10	0^b^	-
Comorbid anxiety	11	NA	-
CES-D at the day of study (M, SD, min/max)	36.67, 8.04, 19/57	4.2, 3.64, 0/14	-

^a^One participant did not report this information. The calculation was based on 41 participants.

^b^One participant did not report this information. The calculation was based on 40 participants.

^c^Independent sample t-test was used to test significance.

^d^Fisher’s Exact test was used to test significance.

NA–Not applicable

### Speech in noise task

All participants completed tasks of sentence recognition across conditions varying in the degree of linguistic information (high, low, and none): 1-talker masker (1T), reversed 1-talker masker (1T_tr) and speech-shaped noise (SSN).

#### Target sentences

The target sentences were pooled from the Revised Bamford-Kowal-Bench (BKB) Standard Sentence Test [50]. Each BKB sentence (e.g., The BUCKETS HOLD WATER) contains three to four keywords (uppercase words). They were recorded by a female native speaker of American English in a sound-attenuated booth at Northwestern University [51]. Three BKB sentence lists (16 sentences in each list, with 50 keywords for scoring) were used in the current study. All sentences were equated for root-mean-square (RMS) amplitude.

#### Maskers

The 1T and SSN were identical to those described in Chandrasekaran et al. (2015). Briefly, eight female speakers of American English were recorded in a sound-attenuated booth at Northwestern University [52], and produced a total of 240 simple, meaningful English sentences (30 for each speaker; e.g., for dessert he had apple pie) [53]. The 30 sentences from each of the eight speakers were equalized for RMS amplitude and concatenated to form a sentence string without silence between sentences. One of the eight 30-sentence strings was used as the 1T track. To create SSN, a steady-state white noise was filtered to match its spectrum with the long-term average spectrum of the full set of 240 sentences (from all eight speakers). To create the 1T_tr, we reversed the 1T track in time, to reduce the linguistic inference caused by the masker. The three masker tracks were truncated to 50s and equated for RMS amplitude.

#### Mixing targets and maskers

Each of the three BKB sentence lists was mixed with one type of masker. Specifically, each target sentence was mixed with a random sample of the corresponding masker track such that the final stimulus was composed as follows: 500 ms of masker, the target and masker together, and a 500 ms masker. We set the signal-to-noise ratio SNR) at -5 dB (i.e., the noise is 5 dB more intense than the target) to avoid floor and ceiling performances on the basis of previous findings [11,13]. In total, there were 48 stimuli (16 mixed with each of the three types of masker) in the task.

#### Testing procedures

During testing, the stimuli were binaurally presented to participants over Sennheiser HD-280 Pro headphones at a constant level (~70 dB sound pressure level). After each stimulus presentation, the participant was required to type out the target sentence. If they were unable to understand the whole sentence, they were encouraged to report any intelligible words and make their best guess. The order of all the 48 sentences was randomized for each participant.

### Keyword accuracy analysis

As in the majority of studies on SPIN (e.g., [9,11,15]) including Chandrasekaran et al. (2015), participants’ responses from the speech in noise task were scored by whether the keywords were correctly identified or not. To be considered as correct, no morphemes could be added to or deleted from the keywords. Otherwise, the responses were treated as incorrect.

### Speech recognition error analysis

**Fig 2:**
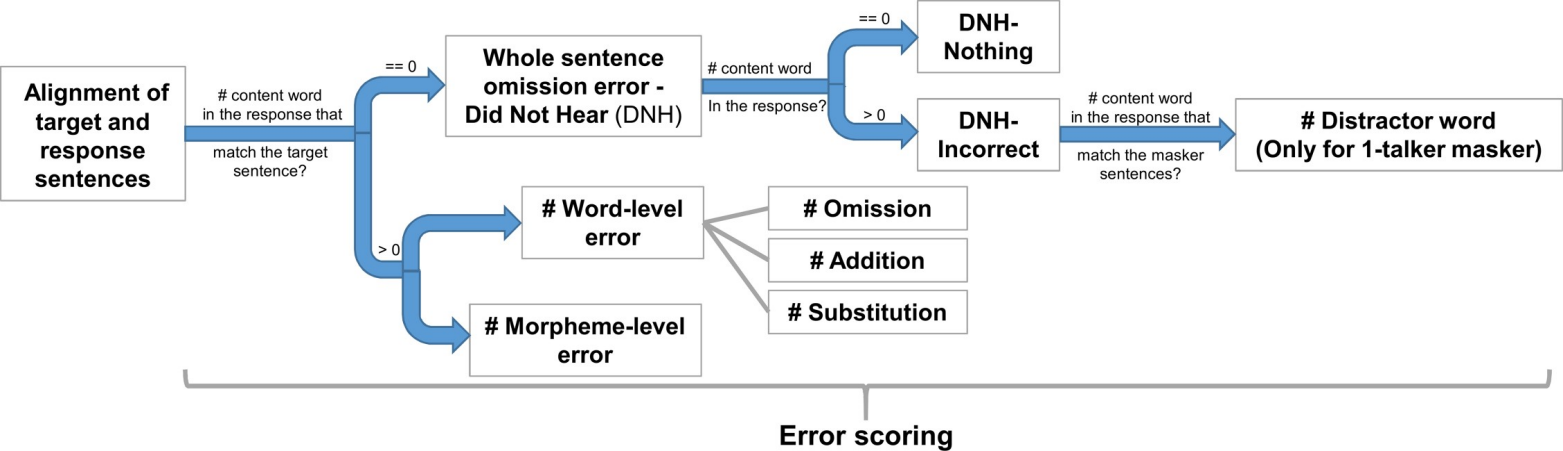
A summary of the speech recognition error analysis.

In addition to keyword accuracy analysis, we also expanded on a prior effort from our group [43] to code the speech recognition errors in participants’ responses from the speech in noise task. The sample code for performing the error analysis is implemented in Python and is publicly available [54]. In the following paragraphs, we provide a detailed description of the speech recognition error analysis. A brief summary of the error analysis is displayed in Fig 2.

For each of the target sentences, participants’ typed response sentences were scored. Rather than scoring only the four keywords in each sentence (which is the gold-standard in assessing SPIN), the entire response sentence was first aligned with the target sentence and then scored for (1) whether the participant produced any content words from the target sentence at all, (2) the word-level errors (e.g., omission of a noun, substitution of a verb) and (3) morpheme-level errors (e.g. tense change, pluralization). The details of these scoring processes are described below. Examples of the various types of errors are shown in Table 2.

**Table 2:** Examples of speech recognition errors.

Error Type	Target Sentence	Aligned Response
DNH_Nothing	he broke his leg	_ _ _ _
DNH-Incorrect	the daughter set the table	_ can go very fast
Omission of content word	a man is turning the faucet	_ _ is turning the faucet
Omission of function word	a man is turning the faucet	_ _ is turning the faucet
Addition of content word	a man is turning the faucet _	a man is turning the faucet trees
Addition of function word	_ father forgot the bread	the father forgot the bread
Substitution of content word	the boy hurried to school	the boy went to school
Substitution of function word	a man is turning the faucet	the man is turning the faucet
Morpheme Error	the daughter set the table	the daughters set the table

Note: errors are marked with underline (for words) or underscore (for gaps)

Sentence alignment was estimated using an adaptation of the Needleman-Wunsch algorithm [55], which uses a global alignment method to infer the best pairwise matches between units in a sequence, in this case, words in the target sentence and response sentence (see Fig 1 in [43] for illustration, available with additional open-source code and dataset in [54]). The algorithm rewards alignment of commonalities (same word) and minimizes the size of the misalignment error (word mismatches or missing words). This approach results in pairings of words or gaps (for missing words), one from the target sentence and one from the response sentence, which can be directly compared.

Our present implementation of the Needleman-Wunsch global alignment algorithm permits the researcher to adjust the weights for different types of matches or mismatches. We adopted the match and mismatch weights from on our previous work with an independent dataset ([43], see [54] for full source code). Correctly matched words were rewarded with +20 because the probability of a correct word appearing in the same place in both target and response sentences by chance is very low (in contrast to other types of sequences with many fewer unique units to select from). Further, we rewarded partial matches (words with Levenshtein distance < = 2, i.e., the number of additions, deletions, or substitutions necessary to match two words. See [56] for full explanation) with +5 to promote correct alignment even when morphological or phonological errors were present. Finally, both mismatches and gaps were penalized at -5. The cumulative result was better scoring of sentences wherein alignment of matched or nearly matched words was consistently preserved while also identifying gaps.

The target and response sentences were tagged as content words (nouns, verbs, adjectives, adverbs) and function words (closed-class) using the Pattern module for Python [57]. Words in the response sentence that did not appear in the Pattern module’s dictionary were replaced with the first suggested spelling substitution if the replacement matched any content word in the target sentence, which allowed for alignment and matching of common typographical errors but rejected any correctly spelled words, such as homophones (consistent with criteria used by human coders in previous studies). At this stage, whole sentence omission errors were identified as “Did Not Hear” (DNH) and removed from further word- and morpheme-level analysis. If a response sentence did not contain any of the content words from the target sentence (regardless of their position in the alignment, and not including forms of the verb “to be”), the trial was marked as DNH. Sentences marked with DNH were further classified to indicate whether the participant transcribed irrelevant content (i.e., content words, but none matching the target; DNH-Incorrect) or simply failed to transcribe any content words at all (DNH-Nothing). Sentences marked as DNH were analyzed as a separate category of error and compared with the masker sentences to determine whether the subject has transcribed the masker content or just entered irrelevant words. DNH-Incorrect sentences were not included in subsequent word- and morpheme-level analyses.

For the remaining trials, the aligned target and response sentences were scored by the script for word-level errors and morpheme-level errors. Word-level errors were aggregated across specific types of omissions, additions, and substitutions: If a given pair of sentences (target + response) contained a word from the target sentence but no word (a gap) from the response sentence, a word-omission was recorded for that trial. If a given pair contained a word from the response sentence but no word (a gap) from the target sentence, a word-addition was recorded for that trial. When two function words in a pair were not identical, a word-substitution was recorded. To evaluate morpheme-level errors, pairs of content words which did not match between target and response were further reduced to their root forms using the Pattern module and compared again. If two content words matched in their root forms but not in the original target and response, a morphemic error was recorded. However, if the words did not match in root form, a word-substitution error (at the word level) was recorded instead.

### Statistical analysis

#### Keyword accuracy

The keyword accuracy data were analyzed with generalized linear mixed-effects logistic regression using the lme4 package [58] in R version 3.2.0 [59] where keyword recognition accuracy (correct or incorrect) was modeled as a dichotomous dependent variable. In the model, fixed effects included the depression group (MDD or control) and masker type (1T, 1T_r, and SSN), and their interactions. To account for baseline differences in speech recognition performance across subjects and sentences, we included by-subject and by-sentence intercepts as random effects. Fixed factors were treated as categorical variables. In the model, the reference levels were the control group and 1T.

We tested the interaction between depression group and masker type by comparing a model with such interaction and the lower level effects to a model with only the lower level effects. We examined the main effects of depression group and masker type by comparing the base model (which only included the random-effects structure) to the same model but with the addition of depression group or noise. Model comparisons were achieved using the likelihood ratio [60]. Post hoc analysis for significant interaction or main effect, if necessary, was carried out by Tukey’s tests using the ‘glht’ function of the multcomp package [61]. Multiple comparisons were corrected using the Benjamini-Hochberg false discovery rate method [62].

#### Speech recognition errors

We calculated five error types: DNH-Nothing, DNH-Incorrect, content word errors, function word errors, and morphemic errors. Specifically, for each masker condition (1T, 1T_tr, or SSN) in individual participants, first, we calculated the proportion of sentences (out of the total number of 16 sentences) that were classified as DNH-Nothing and DNH-Incorrect, respectively. For DNH-Incorrect errors, we further calculated the proportion of content words that were from the masker sentences. We restricted this analysis to the 1T condition because only masker sentences from this condition are intelligible. Second, we calculated the mean number of errors per sentence on content words, function words, and morphemes, respectively. We focused these analyses on the sentences that were not categorized as DNH-Nothing or DNH-Incorrect. For both content and function words, we combined all the three error types: substitution, addition, and omission.

For each of the five error types, the data were analyzed with linear mixed-effects regression using the lme4 package [58] in R version 3.2.0 [59]. In the model, fixed effects included the depression group (MDD or control) and masker type (1T, 1T_tr, and SSN), and their interactions. To account for baseline differences across subjects, we included by-subject intercept as random effects. Fixed factors were treated as categorical variables. In this model, the reference levels were the control group and 1T. We applied approaches similar to those for the keyword accuracy analysis as described above to test the interaction effect and the main effects of depression group and masker type. Descriptive statistics, if reported, represent mean ± standard deviation (*SD*).

## Results

### Keyword accuracy

Descriptively, as shown in Fig 1, the mean accuracy was lower in the MDD group than the control group in the 1T (MDD: 65.2% ± 15.4% vs control: 75.0% ± 11.7%) and 1T_tr condition (MDD: 81.5% ± 7.3% vs. control: 86.3% ± 7.8%), but was comparable between the two groups in the SSN condition (MDD: 73.8% ± 13.3% vs. control: 77.2% ± 11.2%). Further, performance variability was larger in the 1T condition than the two other conditions. The generalized linear mixed-effects logistic regression model yielded significant main effects for depression group [*χ*^*2*^ (1) = 4.285, *p* = 0.039] and masker type [*χ*^*2*^ (2) = 6.305, *p* = 0.043], and of primary interest, a significant interaction between depression group and masker type [*χ*^*2*^(2) = 10.418, *p* = 0.005]. Follow-up analysis revealed that, as shown in Fig 1, word recognition in noise was significantly worse for the MDD group than for the control group in the 1T condition [*β =* -0.644, *SE* = 0.229, *Z* = -2.808, *p* = 0.025]. This depression-related deficit of word recognition was not significant in the 1T_Tr condition [*β =* -0.432, *SE* = 0.236, *Z* = -1.832, *p* = 0.143] or in the SSN condition [*β =* -0.267, *SE* = 0.232, *Z* = -1.15, *p* = 0.375].

Further, we tested the model with the addition of three covariates: currently taking medication (medication: yes or no), currently in therapy or counseling (therapy: yes or no) and co-morbid anxiety (anxiety: yes or no) as covariates. One participant was excluded from this analysis because of missing data on the therapy information. The model was construed as: keyword recognition ~ depression group * masker type + medication + therapy + anxiety + (1 | sentence) + (1 | subject). The inclusion of these covariates jointly did not significantly improve model fit, *χ*^*2*^(3) = 0.785, *p* = 0.853, suggesting that the effects of these covariates were not significant.

### Speech recognition errors

**Fig 3:**
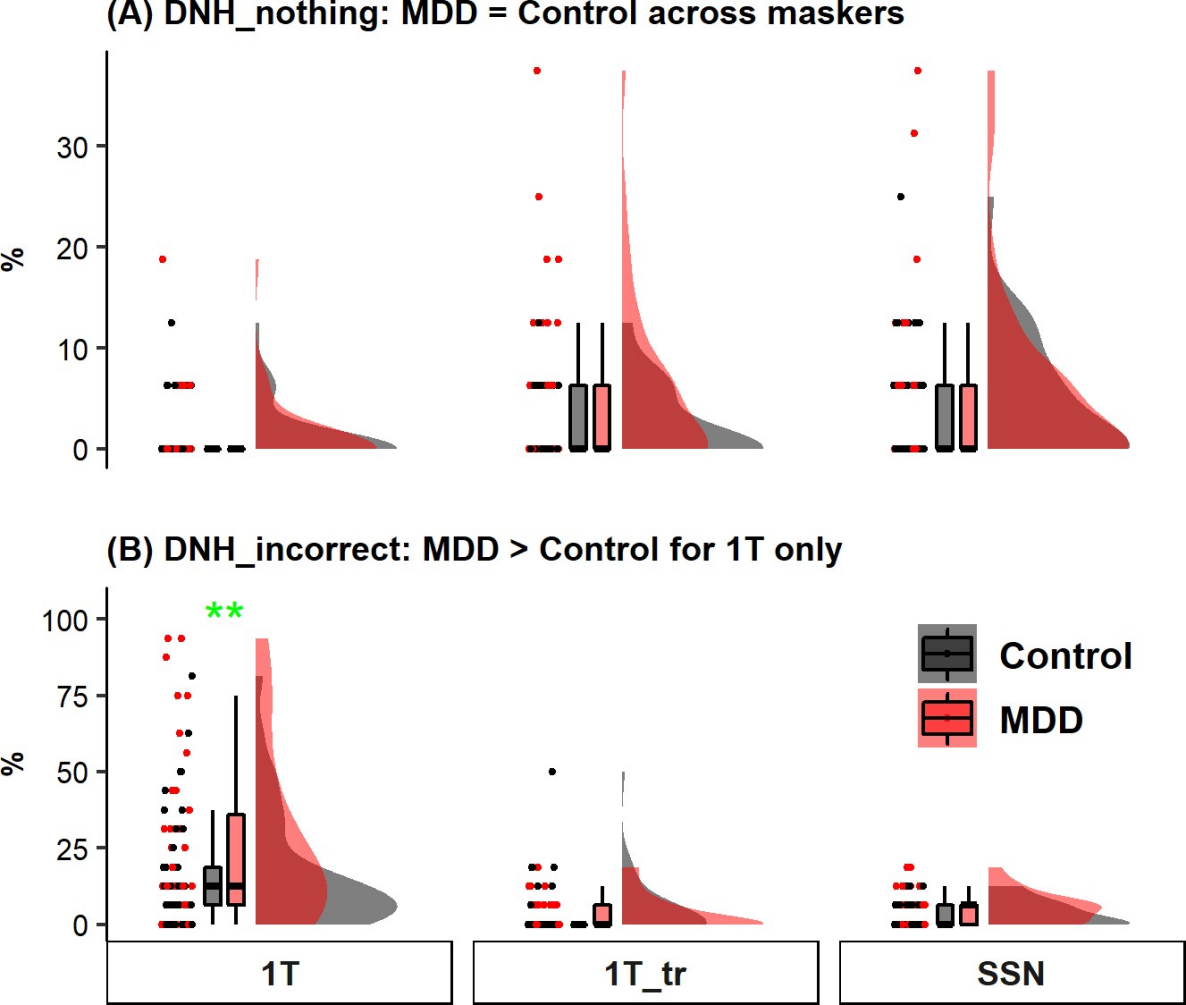
Raincloud plots (from left to right: jittered raw data for all participants, boxplots, and probability distribution of the data) for whole sentence omission errors (i.e., “Did Not Hear”; DNH) from neurotypical controls (black) and participants with MDD (red) across three types of masker: 1T (1-talker masker; left panels), 1T_tr (*reversed* 1-talker masker; middle panels), and SSN (speech-shaped noise; right panels). (A) Proportion of DNH-Nothing errors. This type of error refers to that participants failed to transcribe any content words. (B) Proportion of DNH-Incorrect errors. This type of error refers to that participants transcribed at least one content words but none of them matches the roots for content words from the target sentence. For the boxplots, the boxes and the horizontal line inside show the quartiles (1st to 3rd quartile) and the median, respectively. The whiskers denote 1.5 times the interquartile range. Outliers, defined as cases with values outside the 1.5 interquartile range, were not displayed in the boxplots. ** denote *p* < 0.01.

First, we calculated the proportion of DNH-Nothing errors (Fig 3A) for each condition in individual participants. Descriptively, the mean proportion of DNH-Nothing errors was higher in the MDD group (5.4% ± 8.0%) than the control group (2.1% ± 3.6%) in the 1T_tr condition, but was comparable between the two groups in both the 1T (MDD: 1.0% ± 3.4% vs. control: 1.1% ± 2.8%) and SSN (MDD: 5.1% ± 8.2% vs. control: 4.3% ± 5.8%) conditions. The linear mixed-effects model showed that the main effect of depression group was not significant [*χ*^*2*^ (1) = 2.216, *p* = 0.137]. The main effect of masker type was significant [*χ*^*2*^ (2) = 22.356, *p* < 0.001]. The interaction between depression group and masker type was not significant [*χ*^*2*^(2) = 4.899, *p* = 0.086]. Post hoc analysis for the main effect of masker type revealed that the proportion of DNH-Nothing errors was significantly lower in the 1T condition relative to the other two masker conditions [1T vs. 1T_tr: *β =* 0.0271, *SE* = 0.00774, *Z* = 3.504, *p* < 0.001; 1T vs. SSN: *β =* 0.0361, *SE* = 0.00774, *Z* = 4.672, *p* < 0.001]. There was no significant difference between 1T_tr and SSN conditions [*β =* 0.00904, *SE* = 0.00774, *Z* = 1.168, *p* = 0.243].

Second, we calculated the proportion of DNH-Incorrect errors (Fig 3B) for each condition in individual participants. Descriptively, the mean proportion of DNH-Incorrect errors was higher in the MDD group (26.5% ± 28.1%) than the control group (16.3% ± 19.1%) in the 1T condition, but comparable between the two groups in both the 1T_tr (MDD: 4.4% ± 5.7% vs. control: 3.8% ± 9.3%) and SSN (MDD: 5.2% ± 5.1% vs. control: 3.4% ± 4.4%) conditions. The linear mixed-effects model showed that the main effect of depression group was not significant [*χ*^*2*^ (1) = 3.222, *p* = 0.073]. The main effect of masker type was significant [*χ*^*2*^ (2) = 70.892, *p* < 0.001]. The interaction between depression group and masker type was significant [*χ*^*2*^(2) = 6.835, *p* = 0.033]. Follow-up analysis revealed that the proportion of DNH-Incorrect errors was significantly higher for the MDD group than for the control group in the 1T condition [*β =* 0.102, *SE* = 0.0326, *Z* = 3.117, *p* = 0.003]. However, the MDD vs. control group difference was not significant in the 1T_tr condition [*β =* -0.00388, *SE* = 0.0326, *Z* = -0.119, *p* = 0.97] or in the SSN condition [*β =* 0.0185, *SE* = 0.0326, *Z* = 0.568, *p* = 0.777]. Further, we calculated the proportion of content words in participants’ responses that match the content words from the masker sentences. We conducted this analysis only for the 1T condition. About 70% (MDD: 71.0% ± 25.5%; Control: 72.3% ± 30.3%) of content words in participants’ responses matched those from the maskers. There was no significant difference between the MDD and control group [*t*(58.967) = 0.187, *p* = 0.853].

**Fig 4:**
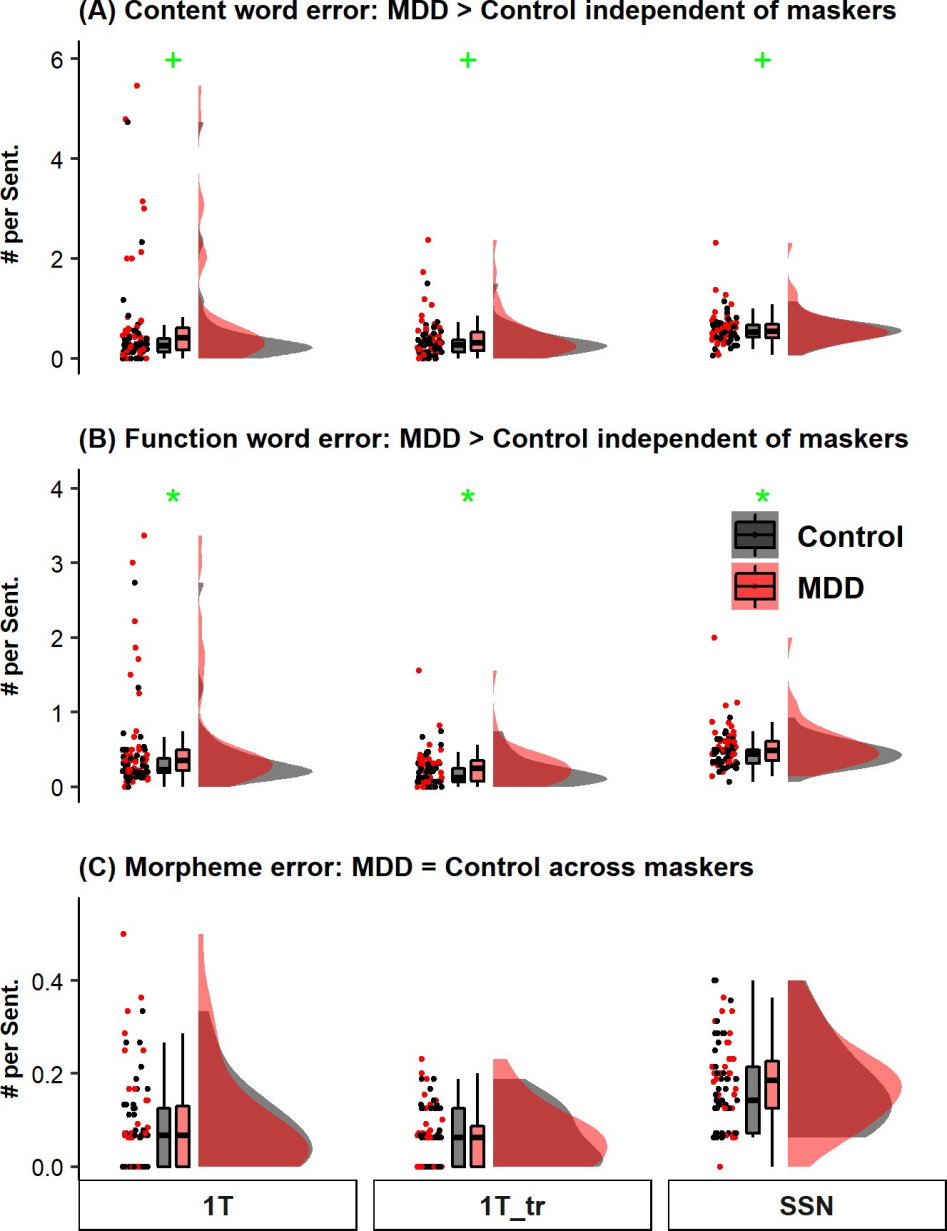
Raincloud plots (from left to right: jittered raw data for all participants, boxplots, and probability distribution of the data) for word- and morpheme-level errors from neurotypical controls (black) and participants with MDD (red) across three types of masker: 1T (1-talker masker; left panels), 1T_tr (*reversed* 1-talker masker; middle panels), and SSN (speech-shaped noise; right panels). (A) The mean number of errors per sentence on content words. This type of error includes substitution, addition, or omission of nouns, verbs, adjectives, or adverbs. (B) The mean number of errors per sentence on function words. This type of error includes substitution, addition, or omission of closed-class word. (C) The mean number of errors per sentence on morphemes. This type of error refers to cases where the content word from participant’s response matched in the root form with the content word in the target sentence but is not the same as the target content word. For the boxplots, the boxes and the horizontal line inside show the quartiles (1st to 3rd quartile) and the median, respectively. The whiskers denote 1.5 times the interquartile range. Outliers, defined as cases with values outside the 1.5 interquartile range, were not displayed in the boxplots. * denotes *p* < 0.05. + denotes *p* < 0.06.

Finally, for the non-DNH errors, the mean number of errors per sentence was calculated for content words (Fig 4A), function words (Fig 4B), and morphemes (Fig 4C), respectively. Descriptively, the mean number of content and function words errors was higher in the MDD group than the control group across the three masker conditions, while the mean number of morphemic errors was comparable between the two groups across the three masker conditions. Separate statistical analysis was applied to the three error types. The linear mixed-effects models showed that, the main effect of depression group was (marginally) significant for content word errors [*χ*^*2*^ (1) = 3.83, *p* = 0.05] and function word errors [*χ*^*2*^ (1) = 5.223, *p* = 0.022], suggesting that these two types of errors were significantly higher for the MDD group than for the control group. The main effect of depression group was not significant for morphemic errors [*χ*^*2*^ (1) = 0.284, *p* = 0.594]. The main effect of masker type was significant for all three error types [content word errors: *χ*^*2*^ (2) = 8.258, *p* = 0.016; function word errors: *χ*^*2*^ (2) = 26.478, *p* < 0.001; morphemic errors: *χ*^*2*^ (2) = 70.533, *p* < 0.001]. The interaction between depression group and masker type was not significant for all three error types [content word errors: *χ*^*2*^ (2) = 2.988, *p* = 0.225; function word errors: *χ*^*2*^ (2) = 2.549, *p* = 0.28; morphemic errors: *χ*^*2*^ (2) = 0.113, *p* = 0.945]. Post hoc analysis for the main effect of masker type revealed that the number of content word errors and function word errors was significantly higher in the 1T and SSN conditions relative to the 1T_tr condition (all *p*s ranging from 6.73 × 10^−6^ to 0.039). The number of morphemic errors was significantly higher in the SSN condition relative to the 1T condition (*p* < 0.001) and the 1T_tr condition (*p* < 0.001). No other comparisons were significant (all *p*s ranging from 0.088 to 0.805).

## Discussion

### Summary of findings

In a clinical population, the current study replicated the effect of depression on SPIN observed in a population with sub-clinical elevated depressive symptoms from Chandrasekaran et al. (2015). Individuals with MDD, relative to neurotypical NH participants, exhibited lower keyword accuracy in conditions with speech maskers (1T), but not in conditions with non-speech maskers (1T_tr or SSN) (Fig 1).

Critically, we applied a speech recognition error analysis approach [43] to analyze error patterns to understand the nature of the depression-related listening condition-specific (i.e., speech maskers) deficit in speech perception. Particularly, we calculated the occurrence rate of errors that a listener transcribed words irrelevant to the target sentences (DNH_Incorrect; Fig 3B) and found that such error type was significantly higher for individuals with MDD than the neurotypical participants in the conditions with speech maskers. In such condition (speech maskers), words from the masker sentences constituted a great proportion (~70%) of the DNH_Incorrect errors. Meanwhile, we did not observe a depression-related listening condition-specific (i.e., speech maskers) pattern for any other error types including content and function word errors and morpheme-level errors (Fig 4A to 4C). Together, these findings are consistent with our prediction that the occurrence rate of errors as a result of interference from the masker sentences would be increased in individuals with MDD (relative to neurotypical controls) in conditions with speech maskers. Mechanistically, the increased interference from the masker sentences may be related to heightened susceptibility to linguistic interference from distracting talkers.

### Increased susceptibility to distracting information in individuals with MDD

Increased susceptibility to distracting information in individuals with MDD has been reported in both behavioral (e.g., [44,45]) and neuroimaging studies (e.g, [46,47]). For example, Lemelin et al. (1997) demonstrated that, in a Stroop color-word test, some individuals with MDD, relative to typical participants, exhibited additional delay (slower response time) in naming the color in the presence of distractor words (relative to the condition without distractors), even if the meaning of the distractors is unrelated to names of color. In an fMRI (functional magnetic resonance imaging) study, Desseilles et al. (2009) revealed that individuals with MDD (relative to control participants) showed increased BOLD (blood oxygenation level-dependent) responses to task-irrelevant visual stimuli in the visual cortices, suggesting less filtering of distracting information. In line with these prior studies, the present study suggests that individuals with MDD (vs. neurotypical controls) are more susceptible to distracting linguistic information that is highly confusable with the target stimuli (i.e., 1T condition).

Note that an early study examined the ability to follow an auditorily presented story in one ear with and without the interference of competing stories from the other ear in a small group (N = 8) of individuals with MDD. Their performance to follow auditory stories was not affected by the presence of the distracting stories [63]. However, the power of this early study [63] may be limited by the small sample size, as well as the potential large variability in the susceptibility to distracting information in individuals with MDD [45]. Therefore, we are inclined to conform to the argument of elevated distractibility to distracting information associated with MDD. Nevertheless, future studies are needed to further elucidate the mechanisms underlying the depression-related listening condition-specific (i.e., speech maskers) deficit in speech perception.

### Analyzing speech recognition errors: Past and current approaches

Prior work has investigated speech recognition errors in phoneme [36,37,40–42] and word [35,38,39] perception tasks. To the best of our knowledge, only one recent study has examined recognition errors for sentence-level materials [34]. Smith and Fogerty (2017) examined two error categories specifically for the sentence keywords across different non-speech noise contexts (speech in SSN and speech periodically interrupted by SSN with 33%, 50% or 66% speech proportion preserved): Whole word error, which includes substitution, addition, and omission of keywords, and part-word error, which includes substitution, addition, and omission of phonemes in the keywords. They found the occurrence rates of whole word and part word errors were higher for speech in SSN and speech interrupted by SSN with the smallest speech proportion preserved (33%) than for speech interrupted by SSN with higher speech proportion preserved (50% and 66%).

Relative to the error analysis approach in Smith and Fogerty (2017), a unique aspect of error analysis approach worth noting is that our approach codes DNH-Incorrect errors (i.e., participants transcribed at least one content words but none of them matches the roots for content words from the target sentence). The coding of DNH_Incorrect errors is meaningful because our design included a condition with speech masker (1T) wherein a listener is likely to report words from the speech masker as the targets. It should be mentioned that the characterization of interference from speech maskers in SPIN tasks has been reported in the literature (e.g., [64,65]). Those studies typically utilized matrix sentences (i.e., closed-set sentences combined from a limited sets of words) as the target and maskers. Unlike the prior work, our approach directly dealt with open-set sentences that are more realistic in daily-life scenarios. Using our approach, we found that the occurrence rate of DNH_Incorrect errors was higher in individuals with depression in the 1T condition. Thus, the error analysis, beyond the keyword accuracy analysis, helps, to some extent, pinpoint the locus of deficit in SPIN associated with depression. It is conceivable that future studies on speech perception can benefit from the combination of keyword scoring analysis and recognition error analysis.

### Implications for SPIN studies with hearing impairment and aging

The study of the independent effect of depression on speech perception, as in the current study, represents a meaningful contribution to the field related to SPIN. As mentioned earlier, a listener’s ability to understand speech, particularly in the presence of interfering talkers, can be independently affected by hearing impairment and aging [19–23]. Interesting, these two factors are suggested to increase risk for depression [24–31]. Hence, considering our finding of the depression-related listening condition-specific deficit in speech perception, we propose the need to understand the extent to which depression exacerbates the difficulty of speech understanding in individuals with HI or older adults. Note that the current study assessed speech perception in certain noise conditions (e.g., a fixed SNR) to avoid floor and ceiling performances, further studies are needed to extend the current findings to a range of noise conditions (e.g., a wide range of SNRs).

### Larger individual variability in speech recognition under speech maskers

Qualitatively, there are larger individual differences in speech recognition performance under speech maskers (1T) relative to non-speech maskers (1T_tr and SSN) (Fig 1). Such observation is consistent with our previous studies (e.g., [11]). As mentioned in the introduction, while speech maskers and non-speech maskers both produce energetic masking (though to a different extent), speech maskers additionally produce informational masking [8,9,15,16]. Speech recognition under informational masking places demands on individual’s executive abilities (e.g., working memory) [11]. Hence, individual variations in executive abilities likely contribute to the larger individual variability in speech recognition under speech maskers.

## Conclusions

We present evidence that individuals with MDD exhibited a listening condition-specific deficit in speech perception under speech maskers. Based on the findings from speech recognition error analysis, we posit that this listening condition-specific deficit may be related to heightened susceptibility to interferences from background talkers. Typical social conversations often transpire in environments with distracting talkers. Such listening condition-specific speech perception deficit associated with MDD could lead to (or exacerbate) social and communicative difficulties in individuals with MDD, which may in turn exacerbate their depressive symptoms [4].
